# HigB1 Toxin in *Mycobacterium tuberculosis* Is Upregulated During Stress and Required to Establish Infection in Guinea Pigs

**DOI:** 10.3389/fmicb.2021.748890

**Published:** 2021-11-30

**Authors:** Arun Sharma, Kalpana Sagar, Neeraj Kumar Chauhan, Balaji Venkataraman, Nidhi Gupta, Tannu Priya Gosain, Nikhil Bhalla, Ramandeep Singh, Amita Gupta

**Affiliations:** ^1^Tuberculosis Research Laboratory, Translational Health Science and Technology Institute, Faridabad, India; ^2^Department of Biochemistry, University of Delhi South Campus, New Delhi, India; ^3^Centre for Innovation in Infectious Disease Research, Education and Training, New Delhi, India

**Keywords:** *Mycobacterium tuberculosis*, HigBA1, toxin antitoxin loci, virulence, stringent response

## Abstract

The extraordinary expansion of Toxin Antitoxin (TA) modules in the genome of *Mycobacterium tuberculosis* has received significant attention over the last few decades. The cumulative evidence suggests that TA systems are activated in response to stress conditions and are essential for *M. tuberculosis* pathogenesis. In *M. tuberculosis*, Rv1955-Rv1956-Rv1957 constitutes the only tripartite TAC (Toxin Antitoxin Chaperone) module. In this locus, Rv1955 (HigB1) encodes for the toxin and Rv1956 (HigA1) encodes for antitoxin. Rv1957 encodes for a SecB-like chaperone that regulates HigBA1 toxin antitoxin system by preventing HigA1 degradation. Here, we have investigated the physiological role of HigB1 toxin in stress adaptation and pathogenesis of *Mycobacterium tuberculosis*. qPCR studies revealed that *higBA*1 is upregulated in nutrient limiting conditions and upon exposure to levofloxacin. We also show that the promoter activity of *higBA*1 locus in *M. tuberculosis* is (p)ppGpp dependent. We observed that HigB1 locus is non-essential for *M. tuberculosis* growth under different stress conditions *in vitro*. However, guinea pigs infected with *higB*1 deletion strain exhibited significantly reduced bacterial loads and pathological damage in comparison to the animals infected with the parental strain. Transcriptome analysis suggested that deletion of *higB*1 reduced the expression of genes involved in virulence, detoxification and adaptation. The present study describes the role of *higB*1 toxin in *M. tuberculosis* physiology and highlights the importance of *higBA*1 locus during infection in host tissues.

## Introduction

Tuberculosis (TB), caused by *Mycobacterium tuberculosis* (*M. tuberculosis*) is a major health concern and infects nearly one-third of the world population. The failure of BCG vaccine to impart protection in adult population and HIV co-infection has negative impact over the control of global TB cases. There is a significant increase in the number of patients infected with the *M. tuberculosis* strain resistant to front-line TB drugs such as isoniazid and rifampicin. Studies have shown that the proportion of TB patients infected with multi-drug resistant strains lies in the range of 4.6–25% (Lange et al., 2019). Control of the spread of drug-resistant TB and eradication of TB is hampered by the limited efficacy of therapeutic approaches against drug resistant *M. tuberculosis* strains and our poor understanding of the strategies used by the pathogen for survival inside the human host. *M. tuberculosis* has emerged as a highly successful intracellular pathogen due to its ability to sense extracellular stimuli and reprogram metabolic pathways that enables it to survive in host tissues under varied stress conditions.

Toxin antitoxin (TA) systems are mostly two component modules that are widely present in the genome of prokaryotes and have been implicated in bacterial stress adaptation, persister formation and virulence (Page and Peti, 2016; Harms et al., 2018; Slayden et al., 2018). TA module encodes a toxin that generally inhibits bacterial growth in a bacteriostatic manner by inhibiting an essential cellular function (Schuster and Bertram, 2013; Kedzierska and Hayes, 2016; Page and Peti, 2016; Harms et al., 2018; Fraikin et al., 2020; Kamruzzaman et al., 2021). The antitoxin component of the TA operon neutralizes the activity of this toxin. Based on the mechanisms for neutralization of toxin by the cognate antitoxin, the TA systems have been classified into eight types (Lobato-Marquez et al., 2016; Choi et al., 2018; Harms et al., 2018; Song and Wood, 2020; Wang et al., 2021). The *M. tuberculosis* genome encodes for majorly Type II TA systems, where the antitoxin forms a tight complex with toxin and abrogates the activity associated with toxin (Pandey and Gerdes, 2005; Ramage et al., 2009; Sala et al., 2014; Tandon et al., 2019). VapBC family that encodes for the VapC toxin and VapB antitoxin is the most abundant subfamily of TA systems in *M. tuberculosis* (Ahidjo et al., 2011). VapC toxins are characterized by the presence of PIN domain and have been shown to inhibit *M. tuberculosis* growth by targeting either tRNA or rRNA or mRNA (Sharp et al., 2012; Cruz et al., 2015; Winther et al., 2016). The inducible expression of VapC toxins inhibits the growth of *M. tuberculosis* or *M. smegmatis* or *E. coli* in a bacteriostatic manner (Ramage et al., 2009; Winther et al., 2016; Agarwal et al., 2018). Several studies have shown that the expression of a “subset” of TA systems is increased in stress conditions such as nutrient deprivation, low oxygen and in macrophages (Ramage et al., 2009; Gupta et al., 2017; Agarwal et al., 2018). Previously, we have shown that deletion of *vapBC*3, *vapBC*4, *vapBC*11, and *vapC*22 in the genome of *M. tuberculosis* impairs its growth in guinea pigs (Agarwal et al., 2018, 2020; Deep et al., 2018). However, parental, Δ*vapC*28 mutant and Δ*vapC*21 strain displayed comparable growth kinetics in guinea pigs and mice, respectively (Agarwal et al., 2018; Sharma et al., 2020). MazF toxins belonging to MazEF TA systems are sequence specific endonucleases that are cumulatively required for *M. tuberculosis* to establish infection in host tissues (Tiwari et al., 2015). RelE toxins belonging to the RelBE TA system have been shown to be individually non-essential for *M. tuberculosis* virulence in mice tissues but contribute to antibiotic tolerance in a drug-specific manner (Singh et al., 2010).

HigBA TA system was originally identified on Proteus vulgaris plasmid, Rts1 with unique gene arrangement as HigA antitoxin is present downstream of HigB toxin (Tian et al., 1996). HigB belongs to RelE subfamily of toxins and cleaves mRNA in a ribosome dependent manner in *V. cholerae*, *P. vulgaris*, and *E. coli* (Christensen-Dalsgaard and Gerdes, 2006; Hurley and Woychik, 2009; Christensen-Dalsgaard et al., 2010). HigBA TA module from *A. baumannii* is expressed during stationary phase and under iron deficient conditions (Armalyte et al., 2018). In *P. aeruginosa*, activation of HigB toxin influences the levels of intracellular c-di-GMP and virulence factors like pyocyanin and pyochelin (Wood and Wood, 2016). Furthermore, HigB toxin also promotes the killing of immune cells by increasing the expression of type III secretion system in ciprofloxacin induced persisters in *P. aeruginosa* (Li et al., 2016; Wood and Wood, 2016; Zhang et al., 2018). Besides other bicistronic TA operons, *M. tuberculosis* encodes for two HigBA TA loci, HigBA2 (Rv2022c-Rv2021c) and HigBA3 (Rv3182-Rv3183). The genome of *M. tuberculosis* also encodes for a tripartite Toxin Antitoxin Chaperone (TAC) system. TAC system comprises of HigB1 toxin (Rv1955), HigA1 antitoxin (Rv1956) and SecB like chaperone (Rv1957). SecB like chaperone prevents HigA1 aggregation and degradation by interacting with Chad like sequences present within HigA1 (Fivian-Hughes and Davis, 2010; Bordes et al., 2016; Guillet et al., 2019). Studies in *E. coli* and *M. smegmatis* have shown that overexpression of HigB1 toxin inhibits bacterial growth which is restored upon co-expression of cognate antitoxin, HigA1 (Gupta, 2009; Ramage et al., 2009). It has been shown that HigB1 and HigA1 are co-transcribed with upstream genes Rv1954A, Rv1954c and downstream gene, Rv1957. The locus comprises of two promoters, the P2 promoter controls the expression of Rv1954A-Rv1957 locus, whereas, the P1 promoter is inducible in DNA damaging conditions and controls the expression of Rv1955-Rv1957 only. Previously, it has also been reported that HigA1 possesses helix-turn-helix motif at the amino-terminus, binds to the motif ATATAGG(N)_6_CCTATAT and represses the expression of Rv1954A-Rv1957 locus (Fivian-Hughes and Davis, 2010). Schuessler et al. (2013) have shown that inducible expression of *higB*1 decreased IdeR and Zur transcript levels and also cleaves tmRNA. Recently, Texier et al. (2021) has shown that ClpXP1P2 protease complex is involved in HigA1 degradation and proposed a model for HigB1 toxin activation.

In the present study, we have performed experiments to investigate the physiological role of HigB1 in *M. tuberculosis*. Here, we report that HigB1 toxin is upregulated in *M. tuberculosis* under nutrient limiting conditions and upon exposure to levofloxacin. Further, we also demonstrate that in comparison to the parental strain, the growth of Δ*higB*1 mutant strain was impaired in guinea pigs. We also observed that reduced tissue damage in lung sections of Δ*higB*1 mutant strain infected guinea pigs in comparison to the sections from guinea pigs infected with the parental strain. The expression of genes involved in virulence, detoxification and adaptation were reduced in the Δ*higB*1 mutant strain in comparison to the wild type strain. Taken together, in this study, we have investigated the role of HigB1 toxin in physiology and pathogenesis of *M. tuberculosis*.

## Materials and Methods

### Culture Conditions, Construction of Δ*higB*1 Mutant and Complemented Strains

The *E. coli* and mycobacterial strains were cultured at 200 rpm, 37°C in Luria Bertani medium and Middlebrook 7H9 medium supplemented with 0.2% glycerol, 0.05% Tween-80 and 1x ADS, respectively. For CFU enumeration, an aliquot was removed at designated time points, diluted 10-folds and plated on Middlebrook 7H11 agar supplemented with 1x OADS plates at 37°C for 3–4 weeks. Unless mentioned, all reagents and chemicals used in the study were purchased from Sigma Aldrich, Merck. *M. tuberculosis higB*1 gene was deleted from the genome of *M. tuberculosis* using temperature sensitive mycobacteriophages as described previously (Bardarov et al., 2002). Briefly, pYUB854Δ*higB*1 construct was prepared via cloning 800bp upstream (F-gaggccttacgtcctggacaccaacgtggtg, R-gtctagaacccatggcggctggatcaggggg) and downstream (F- gaagcttagagccttcggcgacaccccaccga, R-gactagtactcgaaatcagcggtg gctacgtc) regions of *higB*1 gene in cosmid pYUB854 flanking the hygromycin resistance cassette. The recombinant cosmid was digested with *Pac*I and packaged in phagemid, phAE87 using Gigapack III Gold Packaging Extract. The recombinant cosmid was electroporated in *M. smegmatis* to generate high titer temperature sensitive mycobacteriophages. The high titer phages were used to transfect *M. tuberculosis* H37Rv strain to generate Δ*higB*1 mutant strain. The deletion of *higB*1 gene was confirmed by performing whole genome sequencing using the Nextera XT kit and associated protocols on MiSeq (Illumina). The complemented strain was constructed by cloning *higB*1 gene with its upstream region in the integrative mycobacterium expression vector pMV306K. The recombinant pMV306K-*higB*1 was electroporated in Δ*higB*1 mutant strain and transformants were selected on 7H11 agar plates containing hygromycin and kanamycin.

### Real Time Polymerase Chain Reaction Studies

In order to determine *higB*1 and *higA*1 expression levels in disease relevant stress conditions, total RNA was isolated from *M. tuberculosis* H37Rv strain exposed to various stress conditions. These conditions were (i) oxidative stress (5 mM H_2_O_2_), nitrosative (5 mM NaNO_2_, 7H9 medium, pH-5.2), nutritional stress (1x Tris buffer saline with 0.05% Tween 80), isoniazid treatment (10 μg/ml) and levofloxacin treatment (10 μg/ml). In order to measure intracellular expression levels, total RNA was isolated from J774.1 macrophage infected with *M. tuberculosis*. The isolated RNA from different conditions was DNase I treated, cDNA synthesized and qPCR was performed as previously described (Agarwal et al., 2018).

### Promoter Activity Assays

For promoter activity assays, upstream region of *higB*1 was Polymerase Chain Reaction (PCR) amplified and cloned in an EGFP-based promoter reporter vector, pSCK301T3. The recombinant plasmid was electroporated into wild type, Δ*higB*1, Δ*ppk*1, and Δ*rel*A strains and transformants were selected on Middlebrook 7H11 medium supplemented with kanamycin and hygromycin. For measurement of promoter activity, strains were cultured in MB7H9 medium till different stages of growth and fluorescence measurements were determined using a Spectramax M5 plate reader (Molecular devices, Inc., United States) with excitation at 490 nm and emission at 520 nm.

### *In vitro* Stress and Drug-Persistence Experiments

*In vitro* growth characteristics of parental, Δ*higB*1 mutant and complemented strains (Δ*higB*1-CT) was determined in MB7H9 medium by measuring OD_600 nm_ at regular intervals. For *in vitro* stress experiments, early-log phase cultures of various strains were exposed to either 5 mM H_2_O_2_, 5 mM NaNO_2_, 0.25% SDS, or 2.5 mg/ml lysozyme for 24 or 72 h. For nutritional starvation, early-log phase cultures of various strains were harvested, washed and resuspended in 1x tris buffered saline containing 0.05% Tween 80 (1x TBST-80) for either 7 or 14 days. The biofilm formation and colony morphology experiments for various strains were performed as previously described (Agarwal et al., 2018; Arora et al., 2018). For *in vitro* drug-susceptibility assays, mid-log phase cultures of various strains were exposed to drugs that possess a different mechanism of action. The drugs used in the study were isoniazid (cell wall inhibitor), rifampicin (transcription inhibitor), and levofloxacin (replication inhibitor).

### Animal Experiments

*In vivo* guinea pig experiments were performed as per the guidelines provided by Committee for the Purpose of Control and Supervision of Experiments on Animals (CPCSEA, Govt of India). The experiments were conducted with prior permission of the institutional animal ethics committee of University of Delhi, South Campus. Single cell suspension was prepared from mid-log phase cultures of various strains and aerosol infection was performed using Madison aerosol exposure chamber. The aerosol infection resulted in implantation of 50–100 bacilli in lung tissues at day 1 post-infection. The bacterial loads and histopathology analysis were performed at day 28 and day 56 post-infection. For CFU enumeration, both lungs and spleens were homogenized in 2 ml saline and 100 μl of 10.0-fold serial dilutions was plated on MB7H11 plates in duplicates. The upper left lobe of infected animals was fixed with 10% formalin and stained with hematoxylin and eosin for histopathology analysis as described previously (Singh et al., 2013, 2016).

### Microarray Experiments

For gene expression profiling, total RNA was isolated from wild type H37Rv, Δ*higB*1 mutant and complemented strains as previously described (Singh et al., 2013). The isolated RNA was treated with DNase I (Thermo Fischer, United States) and quantified using Nanodrop 2000c spectrophotometer (Thermo Scientific, United States). The purity and integrity of RNA samples were checked on Agilent 2100 Bio analyzer (Agilent Technologies Inc., United States). Further, 25 ng of RNA was amplified and labeled using Low input Quick Amp WT Labeling kit (Agilent Technologies, United States) as described previously (Venkataraman et al., 2014). The labeled cRNA was purified using RNeasy columns (Qiagen, United States) and total yields were quantified on Nanodrop 2000c spectrophotometer. The hybridization was performed using Gene expression hybridization kit as per manufacturer’s recommendation (Agilent Technologies, United States). Hybridizations were performed in triplicates. The slides were washed after hybridization as per manufacturer’s instructions and scanned using the Agilent Microarray Scanner at a resolution of 5 μM. The settings used for scanner were: Agilent HD_GX_1 color (61 × 21.6 mm), TIFF 20-bit, Photomultiplier tube (PMT) gain 100%. The scanned image was analyzed using Agilent Feature Extraction software (v10.5). The raw data obtained from microarray experiment was normalized and analyzed using GeneSpring GX v.11.5 software. Normalization of the raw data was performed by taking the 50th percentile for each sample. Baseline correction was applied to the median of all samples. The normalized data has been submitted to NCBI’s Gene Expression Omnibus database (GEO) and can be queried via accession number GSE179403. The differential expression analysis of samples was performed. Genes that showed a twofold or higher change with a *P value* of < 0.05 (unpaired Student *t*-test) were considered to be differentially expressed. Gene Ontology and Pathway analysis of differentially expressed genes was done using DAVID tool^1^ and Panther Classification system^2^. Functional and Protein Interaction Network was performed using StringDB^3^. Clustering of the biologically enriched genes was done using Heatmapper online tool^4^. Gene regulatory network of enriched pathways and genes was performed using Pathreg algorithm (Theomics International Pvt Ltd, Bangalore, India) and visualized using Cytoscape V2.8.3.

### Statistical Analysis

Statistical analysis and generation of graphs was done using Prism 8 software (Version 8.4.3; GraphPad software Inc, CA, United States). Differences between groups were compared using two-tailed *t*-test and were considered significant at *P-value* of < 0.05. The David analysis was also as per the statistical criteria.

## Results

### Deletion of *higB*1 Doesn’t Alter the *in vitro* Characteristics of *Mycobacterium tuberculosis*

**FIGURE 1:**
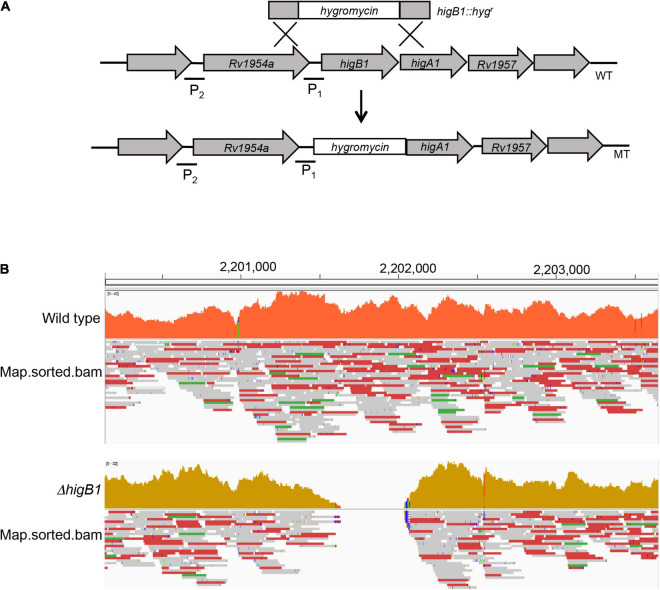
Illustrative representation of *higBA*1 operon in the wild type and mutant strain of *M. tuberculosis.* **(A)** The open reading frame of the *higB*1 gene in the genome of *M. tuberculosis* was replaced with the hygromycin resistance gene using temperature sensitive mycobacteriophages. P1 and P2 stands for promoter region 1 and promoter region 2, respectively. **(B)** The replacement of *higB*1 open reading frame by hygromycin cassette in the Δ*higB*1 mutant strain was confirmed by the whole genome sequencing.

**FIGURE 2:**
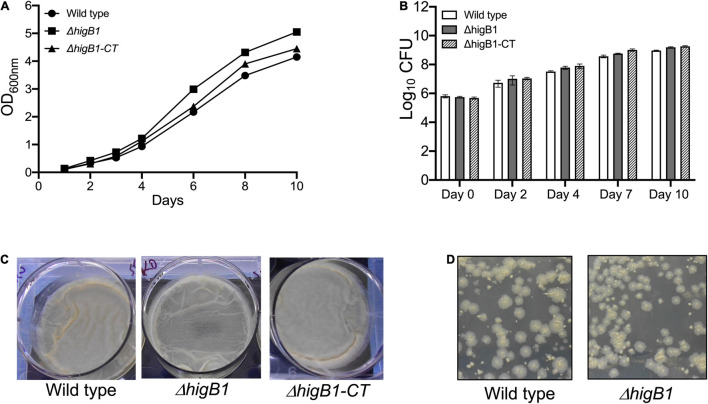
Deletion of HigB1 does not affect *in vitro* growth of *M. tuberculosis*. **(A,B)** The growth patterns of various strains were compared by measuring absorbance at 600 nm **(A)** and CFU enumeration **(B)**. The biofilm formation **(C)** and colony morphology **(D)** was performed as described in section “Materials and Methods.” The data presented shown in **(A,C,D)** panels is representative of two independent experiments. The data shown in panel **(B)** is mean ± SE of Log_10_ CFU obtained from two independent experiments performed in duplicates.

*Mycobacterium tuberculosis higBA*1 locus is unusual as the toxin (HigB1, Rv1955) and antitoxin (HigA1, Rv1956) are co-transcribed along with the upstream gene, Rv1954A and the downstream gene Rv1957 (Cole et al., 1998; Figure 1A). Previously, it has been shown that SecB regulates the activity of HigBA1 locus as it prevents aggregation and degradation of HigA1 antitoxin (Sala et al., 2013; Bordes et al., 2016). It has also been shown that HigB1 overexpression results in growth arrest in *M. tuberculosis* and *E. coli* (Gupta, 2009; Schuessler et al., 2013). In order to determine the role of HigB1 protein in *M. tuberculosis* physiology, Δ*higB*1 mutant strain was constructed using temperature sensitive mycobacteriophages as described in Materials and Methods (Figure 1A, Bardarov et al., 2002). The generation of Δ*higB*1 mutant strain was validated by PCR (data not shown) and whole genome sequencing. As shown in Figure 1B, no sequence reads aligning to the Rv1955 region were obtained in the mutant genome compared to the wild type H37Rv strain genome, confirming that the Rv1955 (*higB*1) sequence was absent in the mutant strain. Further, we did not identify any other secondary mutations in the genomic DNA sequence of the mutant strain. For construction of complemented strain, pMV306K-*hig*B1 was electroporated into Δ*higB*1 mutant strain. Next, the growth patterns of various strains were measured *in vitro* in liquid medium. We did not observe any significant differences in the growth patterns of various strains by measuring either absorbance or bacterial numbers at regular intervals (Figures 2A,B). The bacterial counts of H37Rv, Δ*higB*1 mutant and complemented strains after 10 days were ∼ 9.2 × 10^8^, 1.56 × 10^9^, and 1.81 × 10^9^, respectively (Figure 2B). In *Pseudomonas aeruginosa*, excess of HigB toxin has been shown to reduce the production of various virulence factors and biofilm formation (Wood and Wood, 2016). TA systems have also been implicated in biofilm formation and quorum sensing (Ren et al., 2004; Wang et al., 2011; Wang and Wood, 2011; Sun et al., 2017; Fu et al., 2018). Next, the ability of wild type, mutant and complemented strains to form biofilms was compared (Figure 2C). We observed that wild type, Δ*higB*1 mutant and Δ*higB*1 complemented strain were comparable in their ability to form biofilms *in vitro* (Figure 2C). Also, the colony morphology of the Δ*higB*1 mutant strain was similar to that observed for the parental strain on Middlebrook 7H11 medium (Figure 2D).

### Differential Expression of HigBA1 Locus in Stress Conditions and Its Regulation by RelA Gene Product in *Mycobacterium tuberculosis*

**FIGURE 3:**
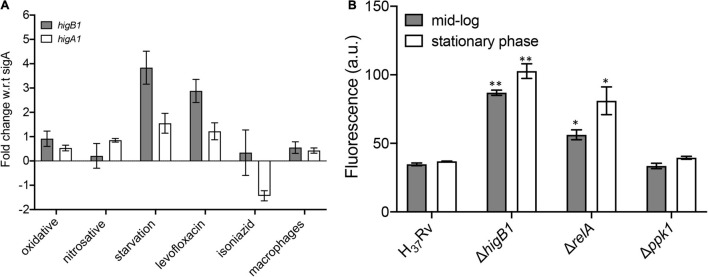
qPCR studies to determine the relative levels of *higB*1 and *higA*1 in different stress conditions. **(A)** The transcript levels of *higB*1 and *higA*1 were determined using gene specific primers as described in section “Materials and Methods.” The data obtained was normalized to the levels of *sigA*, housekeeping gene and shown as mean ± SE obtained from three independent experiments. **(B)** Promoter activity assays. The promoter activity was measured in various strains at different stages of growth as described in section “Materials and Methods.” The data shown in mean ± SE of promoter activity obtained in various strains. Statistically significant differences were observed for the indicated groups, ^∗^*P* < 0.05, *^∗∗^P* < 0.01.

Previous studies have shown that the TAC operon in *M. tuberculosis* is upregulated upon exposure to DNA damaging agents, heat shock, nutritional stress and low oxygen conditions (Betts et al., 2002; Stewart et al., 2002; Rand et al., 2003; Rustad et al., 2008). We also determined the relative levels of *higB*1 toxin and *higA*1 antitoxin by qPCR using gene specific primers after exposure to different *in vitro* stress conditions as described in Materials and methods. In concordance with previous studies, we observed that the expression of *higB*1 was increased by ∼3.8-fold in *M. tuberculosis* upon exposure to nutritional stress. In contrast, the transcript levels of *higA*1 were increased by 1.5-fold in nutritionally starved growth conditions (Figure 3A). No differences in the transcript levels of *higB*1 and *higA*1 was observed after exposure to either oxidative or nitrosative stress (Figure 3A). As shown in Figure 3A, the transcript levels of *higB*1 were also increased by ∼2.8-fold upon exposure to levofloxacin. The expression of *higA*1 remained unchanged in levofloxacin treated *M. tuberculosis* cultures. Further, we also measured the transcript levels of *higB*1 and *higA*1 in macrophages at 24 h post-infection. As shown in Figure 3A, we did not observe any significant changes in the transcript levels of *higB*1 and *higA*1 in *M. tuberculosis* infected macrophages.

Studies have shown that bacteria adapt to nutrient limiting conditions by changing the transcriptome profile to support its prolonged survival (Rohde et al., 2012). The change in transcription profiles in bacterial pathogens is associated with the synthesis of two intracellular alarmones guanosine 5′,3′ bispyrophosphate (ppGpp) and guanosine pentaphosphate (p)ppGpp. In bacteria, the (p)ppGpp cellular levels are regulated by the enzymatic activities of RelA (alarmone synthetase) and SpoT (alarmone synthetase and hydrolase) (Ronneau and Hallez, 2019). The genome of *M. tuberculosis* encodes for a single RelA which is responsible to maintain the cellular pools of (p)ppGpp alarmone (Primm et al., 2000). Studies have shown that RelA protein from *M. tuberculosis* is essential for its long-term survival under starvation and to establish infection in mice tissues (Primm et al., 2000; Weiss and Stallings, 2013). (p)ppGpp levels also regulates the intracellular levels of inorganic polyphosphate (PolyP). The levels of PolyP in bacteria pathogens are regulated by the polyphosphate kinase 1 (PPK-1), Exopolyphosphatases and Polyphosphate kinase 2 (PPK-2). Dysregulation in PolyP levels is associated with attenuation of various intracellular pathogens in animal models (Kornberg et al., 1999; Singh et al., 2013). The (p)ppGpp alarmone and PolyP levels are known to accumulate during stress conditions and these molecules regulate bacterial stress response specifically under nutrient starvation. As *higBA*1 locus was simultaneously induced when H37Rv *M. tuberculosis* was exposed to nutritional limiting conditions, we further analyzed the promoter activity of *higBA*1 locus in Δ*relA* and Δ*ppk*1 mutant strains. For promoter activity assay, pSCK301T3, harboring eGFP downstream of the *higBA*1 promoter region was electroporated in either wild type or Δ*higB*1 or Δ*relA* or Δ*ppk*1 strains and fluorescence was determined at mid-log and stationary stages of growth for various strains. We noticed, that in comparison to the wild type strain, the promoter activity was increased by ∼2.0-fold in stationary phase cultures of Δ*relA* strain of *M. tuberculosis* (Figure 3B, ^∗^*P* < 0.05). Further, 1.6-fold increase in promoter activity was also seen in mid-log phase cultures of Δ*relA* strain as compared to the wild type strain (Figure 3B, *^∗^P* < 0.05). As shown in Figure 3B, we noticed that in comparison to the parental strain, the promoter activity was increased by 2.5-fold and 2.7-fold in Δ*higB*1 strain during mid-log and stationary phase of growth, respectively (^∗∗^*P* < 0.01). These observations suggested that HigB1 toxin acts as a negative regulator of TAC operon expression. The P2 promoter but not the P1 promoter is reported to be regulated by the HigA1 antitoxin (Fivian-Hughes and Davis, 2010). Our data suggests that the HigA1-HigB1 complex negatively regulates the operon. As shown in Figure 3B, no differences were observed in the activity of the promoter of *higBA*1 TAC operon during different stages of growth between parental and Δ*ppk*1 mutant strain. The observed increase in the promoter activity of *higBA*1 TAC operon in Δ*higB*1 and Δ*relA* mutant strains depicts that both *higBA*1 and *relA* gene products regulate the expression of *higBA*1 promoter.

### HigB1 Loci Is Dispensable for Growth of *Mycobacterium tuberculosis* in Different Stress Conditions

**FIGURE 4:**
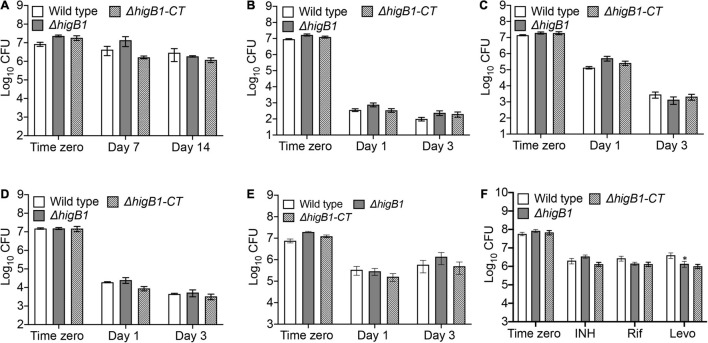
HigB1 is dispensable for *M. tuberculosis* growth in different stress conditions or drugs *in vitro*. The survival of various strains was compared in different stress conditions such as nutritional **(A)** or oxidative **(B)** or nitrosative **(C)** or cell wall disrupting agents, SDS **(D)**, lysozyme **(E)** or after exposure to drugs **(F)**. The data shown in these panels is mean ± SE of Log_10_CFU obtained from two independent experiments performed in either duplicates or triplicates. Statistically significant differences were observed for the indicated groups, ^∗^*P* < 0.05.

In order to survive, the pathogen should be able to sense, adapt and respond to exogenous stress conditions (Fang et al., 2016; Flint et al., 2016). *M. tuberculosis* possess the unique ability to adapt to different environmental conditions inside host tissues during infection. TA systems are generally considered as stress responsive elements as they are differentially regulated under various stress conditions. Under specific stress condition, antitoxin protein is degraded by cellular proteases and free toxin can inhibit the bacterial growth by targeting the essential cellular processes which further facilitate the bacterial survival under these conditions. Previous studies have shown that TAC operon of *M. tuberculosis* is significantly induced in response to heat shock, nutritional starvation, hypoxia and persistence (Betts et al., 2002; Stewart et al., 2002; Rand et al., 2003; Rustad et al., 2008). In concordance, we also observed that the transcripts of *higB*1 were increased upon exposure to nutritional stress and levofloxacin, therefore, we next investigated the role of HigB1 toxin in the *M. tuberculosis* stress adaptation and drug tolerance. We compared the survival of wild type, Δ*higB*1 mutant and complemented *M. tuberculosis* strains upon exposure to various *in vitro* stress conditions. Despite being upregulated in nutrient limiting growth conditions, we observed that the survival of mutant strain was comparable to the parental strain in these conditions (Figure 4A). Also, in other stress conditions tested, the survival of Δ*higB*1 strain was comparable to that observed for the parental strain (Figures 4B–E). Taken together, we conclude that HigB1 toxin does not influence stress adaptation of *M. tuberculosis in vitro*.

### HigB1 Is Required for the Survival of *Mycobacterium tuberculosis* in the Presence of Levofloxacin *in vitro*

Antibiotic persistence is the ability of the bacterial subpopulation to survive the antibiotic treatment. Bacterial persister population makes the TB treatment more difficult and long. Several genes have been implicated in persister formation including TA systems but the role of TA systems in antibiotics mediated persistence is highly questionable. There have been studies which report that overexpression of toxins results in metabolic shutdown that helps the bacterial subpopulation to persist in the presence of antibiotics (Keren et al., 2004a, b, 2011; Tripathi et al., 2014). In *M. tuberculosis*, it has been shown that MazF toxins (MazF3, MazF6, and MazF9) contribute cumulatively to drug persistence in the presence of levofloxacin and rifampicin (Tiwari et al., 2015). However, deletion of either VapBC3 or VapBC4 or VapBC11 or VapC21 or VapC28 or VapC22 in the genome of *M. tuberculosis* did not contribute to drug persistence *in vitro* (Agarwal et al., 2018, 2020; Deep et al., 2018; Sharma et al., 2020). HigBA1 and HigBA2 TA module were also shown to be overexpressed in *M. tuberculosis* persisters (Keren et al., 2011). In *P. aeruginosa*, overexpression of HigB toxin was shown to increase the bacterial survival by 1000-fold after exposure to ciprofloxacin (Li et al., 2016). In order to determine the role of HigB1 in drug persistence, we compared the survival of various strains upon exposure to drugs with different mechanism of action. In accordance with qPCR results, we observed that Δ*higB*1 mutant strain was susceptible to levofloxacin by 3.0-fold after 7 days of exposure in comparison to the parental strain *in vitro* (Figure 4F, *^∗^P* < *0.05*). As shown in Figure 4F, the deletion of HigB1 did not affect the survival of *M. tuberculosis* upon exposure to either isoniazid or rifampicin. We also observed that both parental and Δ*higB*1 strain displayed comparable MIC_99_ values against isoniazid, rifampicin, levofloxacin and ethambutol (Supplementary Table 1). Taken together, these studies suggest that the mutant strain was more susceptible to killing upon exposure to levofloxacin. However, the phenotype was not completely restored in the complemented strain.

### HigB1 Toxin Is Essential to Establish *Mycobacterium tuberculosis* Infection in Guinea Pigs

**FIGURE 5:**
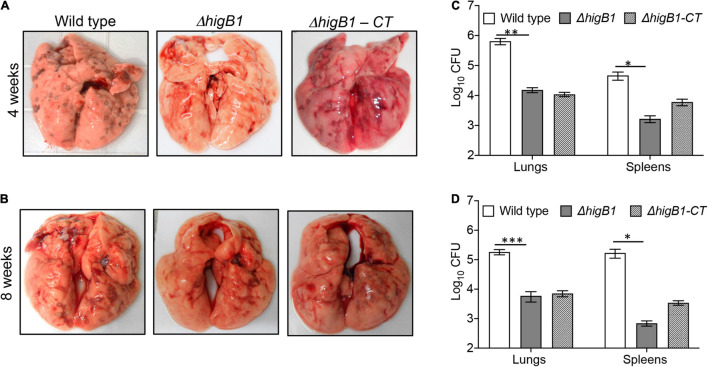
HigB1 locus is important for *M. tuberculosis* pathogenesis in guinea pigs. **(A,B)** This panel depicts representative lung images from guinea pigs infected with either wild type or Δ*higB*1 mutant or *higB*1 complemented strains at 4 weeks **(A)** and 8 weeks **(B)** post-infection. **(C,D)** This panel depicts the lungs and splenic bacillary loads in guinea pigs infected with various strains at 4 weeks **(C)** and 8 weeks **(D)** post-infection. The data shown in this panel is mean ± SE of Log_10_CFU obtained from 6 or 7 animals per group per time point. Statistically significant differences were observed for the indicated groups, ^∗^*P* < 0.05, *^∗∗^P* < 0.01, and *^∗∗∗^P* < 0.001.

Based on the *in vivo* growth phenotype, *M. tuberculosis* strains have been classified as severe growth *in vivo* (sgiv) or growth *in vivo* (giv) or persistence (per) or altered pathology mutants (Hingley-Wilson et al., 2003). TA systems have been implicated in bacterial pathogenesis. We have previously reported that MazF toxins (MazF3, MazF6, and MazF9) contribute to *M. tuberculosis* pathogenesis (Tiwari et al., 2015). Also, in comparison to parental strain, deletion of either *vapBC*3 or *vapBC*4 *or vapBC*11 *or vapC*22 attenuates the growth of *M. tuberculosis* in guinea pigs (Agarwal et al., 2018, 2020; Deep et al., 2018). Recently, it has been reported that deletion of *higB* toxin reduces the virulence of *E. piscicida* in fish tissues (Xie et al., 2021). We next investigated the role of HigB1 in *M. tuberculosis* virulence using guinea pig model of infection. The animals were infected with either parental or Δ*higB*1 mutant or complemented *M. tuberculosis* strains via aerosol route and disease progression was determined during acute (28 days) and chronic stage (56 days) of infection. In concordance with earlier reports, we observed discrete lesions in lung tissues of wild type strain infected guinea pigs (Figures 5A,B). In comparison, significant fewer number of lesions were seen in Δ*higB*1 mutant strain infected guinea pigs. The bacterial counts in the lungs of wild type strain infected animals was log_10_ 5.81 and log_10_ 5.27 at 28 days and 56 days post-infection, respectively (Figures 5C,D). We observed that in comparison to wild type strain, the growth Δ*higB*1 mutant strain was impaired in lung tissues by ∼ 42.0-fold and 31.0-fold, respectively, during acute and chronic stage of infection (Figures 5C,D, ^∗∗^*P* < 0.01 and ^∗∗∗^*P* < 0.001). The *in vivo* growth defect for Δ*higB*1 mutant strain was more prominent at chronic stage specifically in spleens of infected animals. In concordance with the lung data, the bacterial numbers in spleens of parental strain and Δ*higB*1 mutant strain infected guinea pigs was log_10_ 4.6 and log_10_ 3.22, respectively at 4 weeks post-infection (Figure 5C, ^∗^*P* < 0.05). The reduction in splenic bacillary loads of Δ*higB*1 infected animals increased to ∼242.0 folds after 56 days post-infection (Figure 5D, ^∗^*P* < 0.01). The complementation of Δ*higB*1 mutant strain only partially restored the growth defect in spleens of guinea pigs at both time points (Figures 5C,D). Taken together, these observations suggest that TAC locus is required to establish chronic stage of infection in guinea pigs.

**FIGURE 6:**
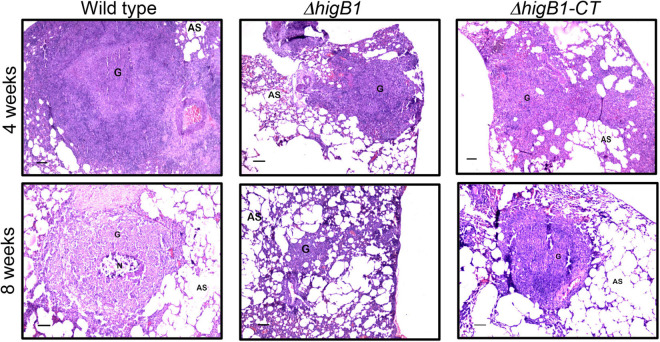
Histopathological analysis of the lung sections obtained from the guinea pigs infected with various strains. The representative photomicrographs of the H&E stained sections (40x magnification) obtained from guinea pigs infected with various strains at 28- and 56-days post-infection. Scale bar – 100 microns.

Further, we performed histopathology analysis of tissue sections obtained from lungs of guinea pigs infected with various strains of *M. tuberculosis* at both 4- and 8-weeks post-infection. In concordance with CFU enumeration data, tissue damage was significantly decreased in the tissue sections from Δ*higB*1 mutant infected guinea pigs. In comparison, the tissue sections from guinea pigs infected with the parental *M. tuberculosis* strain showed heavy tissue damage in both acute and chronic phase of infection (Figure 6). As shown in Figure 6, cellular infiltration of lymphocytes and macrophages was seen in the sections from animals infected with the wild type strain. In comparison, lung sections from Δ*higB*1 strain infected guinea pigs displayed more alveolar space and less damage of lung parenchyma (Figure 6). At 8 weeks post-infection, necrotic areas were present within granulomas signifying extensive tissue damage in sections from parental strain infected guinea pigs (Figure 6). In concordance with CFU data, no necrosis was seen in sections from animals infected with Δ*higB*1 mutant strain at 56 days post-infection. We observed normal lung parenchymal space in tissue sections from guinea pigs infected with the Δ*higB*1 mutant strain (Figure 6). In Δ*higB*1 complemented strain infected guinea pigs, intermediate levels of tissue damage were observed. Overall, *in vivo* CFU enumeration and histopathological analysis demonstrates the importance of HigB1 in establishment of successful *M. tuberculosis* infection in the guinea pigs.

### Global Transcriptome Profiling of Δ*higB*1 Mutant Strain of *Mycobacterium tuberculosis*

**FIGURE 7:**
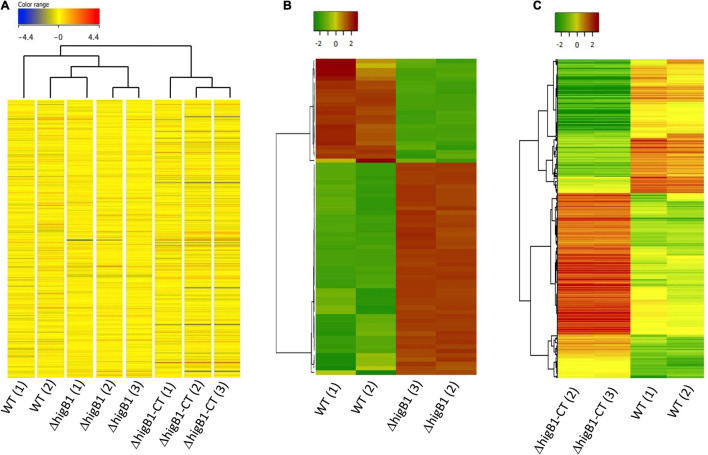
Global transcriptome profile of H37Rv, Δ*higB*1 mutant and complemented strains through microarray analysis. **(A)** The hierarchical cluster analysis, representing the gene expression profile of all genes in sample replicates. Expression values are color coded based upon the extent of expression where red color indicates high level of expression and blue color indicates low level of expression. **(B,C)** The Unsupervised hierarchical clustering of differentially expressed genes in *ΔhigB1* mutant vs. wild type strain **(B)** or complemented strain vs. wild type **(C)**. Expression values are color coded based upon the extent of expression where red color indicates high level of expression and green color indicates low level of expression. The clustering was done using Pearson distance algorithm with average linkage rule.

In order to gain mechanistic insights for the attenuated phenotype of Δ*higB*1 mutant strain of *M. tuberculosis*, we performed microarray experiments to compare the global transcriptome profile of H37Rv, Δ*higB*1 mutant and complemented strain. For microarray experiments, total RNA was isolated from mid-log phase cultures of the strains in either duplicates or triplicates. Using a cut-off value of twofold and *P-value* < 0.05, the fold change was calculated for the genes differentially expressed in the Δ*higB*1 mutant vis-à-vis the wild type H37Rv (Table 1). As expected, *higB*1 transcript levels were reduced by 9.67-fold in the mutant strain (Table 1). Also, the transcript levels of *higA*1 were reduced by 2.18-fold in the mutant strain (Table 1). The transcript levels of Rv1957 did not show any significant change in the mutant strain. The transcript levels of *higB*1 were increased by ∼5.32-fold in complemented strain in comparison to the wild type strain (Supplementary Table 2). Also, there was only a marginal increase in the transcript levels of *higA*1 and *Rv1957* in the complemented strain in comparison to the wild type strain. However, the differential expression of genes observed in the mutant strain was not fully restored in the complemented strain. Unsupervised hierarchical clustering of the samples also showed that the profiles obtained in complemented strain clustered separately from the profiles obtained in the wild type strain (Figures 7A,C). This finding is in concordance with the guinea pig data wherein we did not observe complete restoration of the attenuated phenotype in the complemented strain. It is important to mention here that the complemented strain harbors the integrative pMV306K:*higB1* while the H37Rv wild type and *higB*1 knockout strain lack this site-specific integrative plasmid. To the best of our knowledge, there is no evidence of interference of this plasmid in the gene expression profiles of *M. tuberculosis*.

**TABLE 1:** Differentially expressed genes in *ΔhigB1* mutant vs. wild type strain.

**Gene Id**	**Symbol**	**Fold change**	**Regulation**	**Gene name**
Rv1955	Rv1955	9.67	Down	Toxin HigB
Rv0341	*iniB*	8.65	Down	Isoniazid inducible protein IniB
Rv0914c	Rv0914c	3.38	Down	Lipid carrier protein or keto acyl-CoA thiolase
Rv3139	*fadE24*	3.04	Down	Acyl-CoA dehydrogenase
Rv1779c	Rv1779c	2.97	Down	Integral membrane protein
Rv1057	Rv1057	2.88	Down	Conserved hypothetical protein
Rv0440	*groEL2*	2.81	Down	Molecular chaperone GroEL
Rv0251c	*hsp*	2.56	Down	Heat shock protein
Rv1854c	*ndh*	2.37	Down	NADH dehydrogenase
Rv0118c	*oxcA*	2.34	Down	Oxalyl-CoA decarboxylase OxcA
Rv2428	*ahpC*	2.31	Down	Alkyl hydroperoxide reductase subunit AhpC
Rv3086	*adhD*	2.31	Down	Alcohol dehydrogenase D
Rv3854c	*ethA*	2.24	Down	Monooxygenase EthA
Rv0079	Rv0079	2.23	Down	Unknown protein
Rv0311	Rv0311	2.19	Down	Unknown protein
Rv3084	*lipR*	2.19	Down	Acetyl-hydrolase LipR
Rv1956	Rv1956	2.18	Down	Antitoxin HigA
Rv2729c	Rv2729c	2.16	Down	integral membrane protein
Rv3016	*lpqA*	2.15	Down	Lipoprotein LpqA
Rv2429	*ahpD*	2.08	Down	Alkyl hydroperoxide reductase AhpD
Rv0697	Rv0697	2.07	Down	Dehydrogenase
Rv0244c	*fadE5*	2.04	Down	Acyl-CoA dehydrogenase FadE5
Rv3087	Rv3087	2.04	Down	Diacyglycerol *O*-acyltransferase
Rv3085	Rv3085	2.02	Down	Short chain type reductase SadH
Rv2987c	*leuD*	5.87	Up	3-isopropylmalate dehydratase small subunit
Rv2989	Rv2989	5.80	Up	Transcriptional regulator
Rv2988c	*leuC*	5.76	Up	3-isopropylmalate dehydratase large subunit
Rv1361c	PPE19	5.00	Up	PPE family protein PPE19
Rv0053	*rpsF*	3.41	Up	30S ribosomal protein S6
Rv2624c	Rv2624c	3.20	Up	Universal stress protein
Rv3135	PPE50	3.16	Up	PPE family protein PPE50
Rv0250c	Rv0250c	3.09	Up	Conserved protein
Rv2631	Rv2631	3.00	Up	Conserved protein
Rv3027c	Rv3027c	2.93	Up	GCN5-like *N*-acetyltransferase
Rv2248	Rv2248	2.81	Up	Hypothetical protein
Rv3136	PPE51	2.71	Up	PPE family protein PPE51
Rv2625c	Rv2625c	2.64	Up	Conserved protein
Rv0651	*rplJ*	2.47	Up	50S ribosomal protein L10
Rv0839	Rv0839	2.46	Up	Hypothetical protein
Rv0054	*ssb*	2.44	Up	Single-strand DNA-binding protein
Rv2959c	Rv2959c	2.42	Up	Methyltransferase
Rv0652	*rplL*	2.35	Up	50S ribosomal protein L7/L12
Rv0346c	*ansP2*	2.31	Up	L-asparagine permease
Rv0714	*rplN*	2.29	Up	50S ribosomal protein L14
Rv3340	*metC*	2.28	Up	*O*-acetylhomoserine sulfhydrylase
Rv0055	*rpsR1*	2.27	Up	30S ribosomal protein S18
Rv0715	*rplX*	2.26	Up	50S ribosomal protein L24
Rv1157c	Rv1157c	2.26	Up	Conserved protein
Rv2244	*acpM*	2.25	Up	Meromycolate extension acyl carrier protein
Rv0056	*rplI*	2.24	Up	50S ribosomal protein L9
Rv2990c	Rv2990c	2.24	Up	Hypothetical protein
Rv3924c	*rpmH*	2.20	Up	50S ribosomal protein L34
Rv2254c	Rv2254c	2.20	Up	Integral membrane protein
Rv1158c	Rv1158c	2.19	Up	Hypothetical protein
Rv0634B	*rpmG2*	2.17	Up	50S ribosomal protein L33
Rv1535	Rv1535	2.17	Up	Unknown protein
Rv0700	*rpsJ*	2.16	Up	30S ribosomal protein S10
Rv2933	*ppsC*	2.13	Up	Phthiocerol synthesis polyketide synthase type I
Rv3128c	Rv3128c	2.10	Up	Conserved hypothetical protein
Rv2165c	*mraW*	2.09	Up	Conserved hypothetical protein
Rv0047c	Rv0047c	2.08	Up	Hypothetical protein
Rv0993	*galU*	2.08	Up	UTP–glucose-1-phosphate uridylyl transferase
Rv0315	Rv0315	2.07	Up	Beta-1,3-glucanase
Rv2007c	*fdxA*	2.07	Up	Ferredoxin
Rv2166c	Rv2166c	2.07	Up	Conserved protein
Rv3714c	Rv3714c	2.05	Up	Hypothetical protein
Rv0717	*rpsN1*	2.02	Up	30S ribosomal protein S14
Rv1014c	*pth*	2.02	Up	Peptidyl-tRNA hydrolase
Rv1815	Rv1815	2.01	Up	Hypothetical protein
Rv2431c	PE25	2.01	Up	PE family protein PE25
Rv3260c	*whiB2*	2.01	Up	Transcriptional regulator WhiB2
Rv3628	*ppa*	2.01	Up	Inorganic pyrophosphatase
Rv0430	Rv0430	2.00	Up	Hypothetical protein

Since one of the triplicates for the mutant strain (*ΔhigB*1-1) and the complemented strain (*ΔhigB*1-CT-1) appeared as an outlier in the hierarchical clustering therefore they were not included in the unsupervised hierarchical clustering analysis of the data (Figures 7B,C). We noticed that the relative transcript levels of 73 genes were differentially regulated in Δ*higB*1 mutant strain compared to the wild type strain (Table 1). Among these, the transcripts of Rv2987c, Rv2988c, Rv2989, and Rv1361c were significantly increased by ∼5.0-fold while the transcript levels of Rv0053, Rv2624c, Rv3135, Rv0250c, and Rv2631 were increased by ∼3.0-fold in Δ*higB*1 mutant strain in comparison to the parental strain (Table 1). Further, we also observed that the transcript levels of ribosomal proteins such as Rv0055, Rv0056, Rv0651, Rv0652, Rv0700, Rv0714, Rv0715, Rv0717, and Rv3924c were increased in the Δ*higB*1 mutant strain as compared to the wild type strain (Table 1). Transcriptional profiling of ribosomal proteins and its associated proteins in Δ*higB*1 mutant strain bore close similarities to that induced by *relE*3 overexpression and exposure to protein translation inhibitors (Boshoff et al., 2004; Singh et al., 2010). This corroborates with the fact that HigB1 is a translation inhibitor. In addition, the transcript levels Rv0315 were upregulated by twofold in Δ*higB*1 mutant strain (Table 1). Rv0315 encodes for an immunostimulatory *M. tuberculosis* antigen which activates the dendritic cells and drives the Th_1_ cell response upon *M. tuberculosis* infection (Byun et al., 2012). The transcripts encoding for Rv3027c (GCN5-related *N*-acetyltransferase), Rv3628 (inorganic pyrophosphatase) and Rv3340 (cystathionine [beta]-lyase) were upregulated by 2. 9-, 2. 0-, and 2.3-fold, respectively, in the Δ*higB*1 mutant strain. We also noticed that transcripts of genes such as Rv2007c, Rv2624c, Rv2625c, Rv2631, and Rv3128c belonging to the DosR regulon were also increased in the mutant strain (Park et al., 2003; Table 1). Among these, Rv2624c has been previously reported to be highly immunogenic antigen as and it has been shown to induce higher levels of IFN-γ and TNFα (Bertholet et al., 2011; Chegou et al., 2012).

**FIGURE 8:**
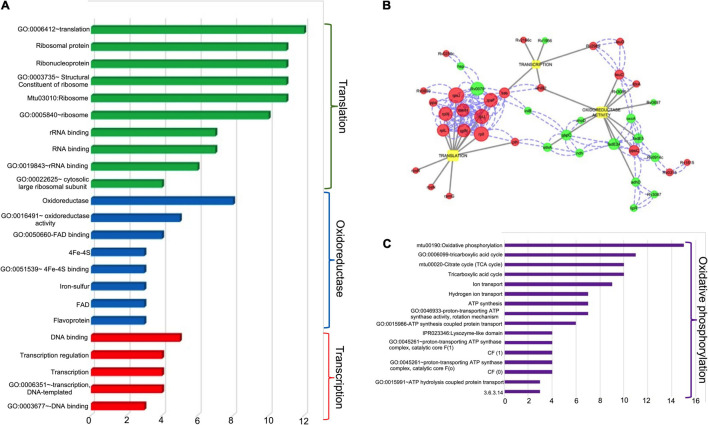
**(A)** Various Gene Ontology (GO) and Pathways enriched for the three major biological functions altered in the *ΔhigB1* mutant in comparison to the wild type strain. *X* axis indicates the number of DEGs under each GO/Pathway. **(B)** The biological regulatory network for the differentially expressed genes. The red color and green circles represent upregulated and down regulated genes in the *ΔhigB1* mutant strain in comparison to the parental strain. The blue dotted lines indicate physical protein-protein interaction and gray solid lines indicate regulation of specific biological functions. The size of the nodes indicates the connectivity score (bigger the size – higher the score). **(C)** Various Gene Ontology (GO) and Pathways enriched for the major biological function altered in the complemented strain in comparison to the wild type strain. *X* axis indicates the number of DEGs under each GO/Pathway.

Further, the transcript levels of 24 genes were down regulated in the mutant strain in comparison to the wild type strain (Figure 7B and Table 1). Among these, 30 and 21% of the proteins belong to the functional category of intermediary metabolism and respiration and virulence, detoxification and adaptation, respectively. The transcript levels of Rv0341 (*iniB*, isoniazid inducible protein), Rv0914c, Rv3139 (keto acyl-CoA thiolase) were reduced by 8.6-fold, 3.3-fold, and 3.0-fold respectively in the Δ*higB*1 mutant strain (Table 1). We also noticed that deletion of *higB*1 in the genome of *M. tuberculosis* reduced the expression of β-propeller gene, Rv1057 by 2.88-fold (Table 1). Previous studies have shown that Rv1057 regulates ESAT-6 secretion and intracellular growth of *M. tuberculosis* (Fu et al., 2018). Also, the transcript levels for Rv3084, Rv3085, Rv3086, and Rv3087 that belong to the acid responsive *mymA* operon (Rv3083–Rv3089) were significantly reduced by ∼2.0-fold in the mutant strain in comparison to the parental strain (Table 1; Singh A. et al., 2003; Singh et al., 2005). The expression of Rv0311, a protein shown to be essential for *M. tuberculosis* to establish extrapulmonary TB infection was also down regulated by ∼2.0 fold in the mutant strain (Be et al., 2008). Further, GO-enrichment analysis was performed using DAVID tool and the most enriched pathways associated with these differentially expressed genes were identified as shown in Figure 8A. These gene sets were then used to create their regulatory network shown in Figure 8B. As evident, the key pathways affected by the *higB*1 deletion were translation, transcription and oxidoreductase family. While a large number of translation-associated proteins and transcription factors were upregulated in the mutant strain, reduced expression of the genes belonging to the oxidoreductase family was observed in the Δ*higB*1 mutant strain. We also observed that in comparison to the parental strain the expression of enzymes involved in translation and transcription pathways was not affected in the complemented strain. These observations indicates that HigB1 expression in the complemented strain restored the expression of proteins belonging to these pathways. However, the expression of enzymes belonging to oxidative phosphorylation pathway was compromised in the complemented strain as compared to the parental strain (Figure 8C).

## Discussion

Chromosomal encoded TA systems are induced in different stress conditions. These have been implicated to help bacteria adapt to different stress conditions by downregulating metabolism and potentiating transition into dormant like stage (Unterholzner et al., 2013; Chan et al., 2016). In addition to slowing down of bacterial metabolism, TA systems are also shown to be essential for persistence and bacterial pathogenesis (Wen et al., 2014; Yang and Walsh, 2017; Alonso, 2021). The large number of TA systems in the genome of *M. tuberculosis* makes it difficult to perceive the involvement of individual TA systems in the pathogen biology. Previous studies have demonstrated that MazF toxins contribute cumulatively and VapBC3, VapBC4, VapBC11, and VapC22 are essential for the *M. tuberculosis* pathogenesis (Tiwari et al., 2015; Agarwal et al., 2018, 2020; Deep et al., 2018). Here, in this study we have investigated the role of HigB1 toxin in *M. tuberculosis* physiology and pathogenesis.

In *M. tuberculosis*, HigB1 cleaves tmRNA, inhibits the growth of bacteria in a bacteriostatic manner and this is abrogated by high levels of HigA1 (Schuessler et al., 2013). The transcript levels of *higB*1 are also increased in *M. tuberculosis* after exposure to nutrient limiting growth conditions and drugs (Betts et al., 2002; Keren et al., 2011). However, its role in bacterial adaptation to these conditions is still not understood. It is still not clear how upregulation of HigB1 to adapt to starvation or exposure to drugs is beneficial to the bacteria or if there is loss of survival/competency in the absence or repression of the toxin. In concordance with previous studies, the transcript levels of *higB*1 were increased in nutrient limiting growth conditions. However, no upregulation of *higB*1 expression was observed in other stress conditions evaluated in the study. The transcript levels of *higB*1 were also upregulated in levofloxacin-treated samples but not after exposure to isoniazid. We observed differential induction of the *higB*1 and *higA*1 belonging to *higBA* TA system upon exposure to levofloxacin and nutritional stress. This might be attributed to differential stability of toxin and antitoxin transcripts in these stress conditions. Similar post-transcriptional regulation of TA system has also been reported in *E. coli* and *M. tuberculosis* in different growth conditions (Korch et al., 2009; Singh et al., 2010; Kasari et al., 2013; Ramirez et al., 2013; Tiwari et al., 2015). Under nutrient limiting conditions, *M. tuberculosis* activates the highly conserved stringent response through guanosine pentaphosphate (p)ppGpp (Primm et al., 2000; Weiss and Stallings, 2013). Previous studies have shown that (p)ppGpp-mediated stringent response in bacteria, in combination with TA activity, can act as a regulated switch to a persistent phenotype (Tian et al., 2017). In *M. tuberculosis*, RelA and PPK-1 are the main enzymes involved in (p)ppGpp and inorganic polyphosphate biosynthesis (Primm et al., 2000; Singh et al., 2013). Since, we observed the increased expression of *higBA*1 locus under nutrient limiting conditions, we determined the promoter activity of TAC locus in parental, Δ*higB*1, Δ*relA* and Δ*ppk*1 mutant strain. The increased promoter activity in Δ*relA* strain suggests that *higBA*1 locus promoter is negatively regulated by (p)ppGpp/relA gene product levels in the cells. Also, we speculate that *higBA*1 locus promoter is negatively regulated by HigB1 either alone or in complex with HigA1 antitoxin as reported previously for other bacterial TA systems (Dienemann et al., 2011; Kang et al., 2017; Nikolic, 2019). This data supports autoregulation and cross regulation between stringent response and TAC system in *M. tuberculosis*.

To further elucidate the role of HigB1 toxin toward *in vitro* and *in vivo* fitness of *M. tuberculosis*, we constructed Δ*higB*1 mutant strain using temperature sensitive mycobacteriophages. The construction of Δ*higB*1 strain was confirmed by Next generation sequencing. The colony morphology and ability to form biofilms was comparable between the parental and Δ*higB*1 mutant strain. Despite being upregulated in nutrient limiting growth conditions, we observed that the survival of both wild type and Δ*higB*1 mutant strain was comparable in nutrient limiting and other stress conditions. In concordance with qPCR results, in comparison to the parental strain, we observed that *ΔhigB*1 strain was compromised for growth upon exposure to levofloxacin. Further, we observed that despite being non-essential *in vitro*, *higB*1 is required for the pathogenesis of *M. tuberculosis* in guinea pigs. Histopathological analysis revealed necrotic granulomatous tissue in lung sections from animals infected with the parental strain. In comparison, normal parenchyma space was seen in sections from animals infected with the Δ*higB*1 mutant strain. The observed attenuation phenotype associated with the mutant strain was more prominent in spleen specifically at chronic stage of infection. The histopathology sections obtain from Δ*higB*1 mutant strain infected guinea pigs appeared more similar to sections from uninfected animals as reported earlier (Singh R. et al., 2003; Patel et al., 2011; Cai et al., 2019). The phenotype associated with Δ*higB*1 mutant strain was similar to that observed for *M. tuberculosis* strains deficient in either CarD or PerM or MymA or Icl1 or PcaA. These strains were also attenuated for growth in spleens and the phenotype was more drastic in chronic stage of infection (Glickman et al., 2000; McKinney et al., 2000; Singh et al., 2005; Weiss et al., 2012; Goodsmith et al., 2015). The success of *M. tuberculosis* as an intracellular pathogen lies in its ability to persist in later stages of infection despite the induction of host adaptive immune response. These observations suggest that HigB1 is important for disease progression and dissemination in guinea pig model of infection.

To gain further mechanistic insights into the attenuation of Δ*higB*1 mutant strain in guinea pigs, we compared the global transcriptome profile of H37Rv, Δ*higB*1 mutant and complemented strains. We observed that the transcripts of ribosomal proteins of both smaller and larger subunits of ribosome were upregulated in the Δ*higB*1 mutant strain. Studies have shown that these ribosomal proteins are able to elicit a strong CD4^+^ immune response that might be associated with the faster clearance of the mutant strain in host tissues (Johnson et al., 2017; Kennedy et al., 2018). In addition to this, expression level of *higA*1 (Rv1956), an adjacent gene of *higB*1 toxin was also reduced in the Δ*higB*1 deletion strain. However, transcripts levels of SecB- chaperone protein Rv1957 was not significantly changed in the mutant strain. Microarray studies revealed that the expression of *higB*1 was restored in the complemented strain by fivefold in comparison to the wild type strain, whereas only marginal change was observed in the levels of *higA*1 and Rv1957. Despite the restoration of *higB*1 levels in complemented strain, expression of other genes such as Rv0311, Rv0315, Rv0341, ribosomal proteins (Rv0651, Rv0652, Rv0700, Rv0714), Rv0914c, Rv1057, and mymA operon (Rv3083-Rv3089) was not restored in the complemented strain. In both cases (Δ*higB*1 knock out and complemented strains) *higB*1 levels were either significantly depleted (9.67-fold) or significantly increased (5.18-fold) in comparison to the parental strain. Previous studies have shown that toxin and antitoxin stoichiometry is important for their autoregulation and activation (Vandervelde et al., 2017; Fraikin et al., 2020). We speculate that changes in intracellular toxin antitoxin ratios in both mutant and complemented strain might be responsible for the observed attenuated phenotype and partial restoration of disease pathology in guinea pigs infected with the *higB*1 complemented strain.

Taken together, we have performed experiments to elucidate the role of HigB1 toxin in *M. tuberculosis* physiology and pathogenesis. We show that HigB1 of *M. tuberculosis* is important to establish infection in guinea pigs. Microarray analysis revealed that deletion of *higB*1 leads to increase in the transcripts of ribosomal proteins and reduction in expression of genes involved in virulence, detoxification and adaptation. This might be responsible for the observed attenuated phenotype of Δ*higB*1 mutant strain. Lack of complementation of the mutant strain could be attributed to altered intracellular ratios of toxin, antitoxin and observed differences in the transcription profiles of wild type and complemented strains. In conclusion, HigB1 is vital for *M. tuberculosis* pathogenesis.

## Data Availability Statement

The datasets presented in this study can be found in online repositories. The names of the repository/repositories and accession number(s) can be found in the article/Supplementary Material.

## Ethics Statement

The animal study was reviewed and approved by University of Delhi South Campus.

## Author Contributions

RS and AG conceived the study and designed the work plan. AS, KS, BV, NG, NC, and TG performed the cloning and microbiology assays. AS, NC, and TG performed the animal experiments. BV isolated the genomic DNA. AG performed the NGS. KS, AG, and AS performed the microarray studies and analysis. NB carried out the analysis of the NGS data. RS, AS, KS, and AG analyzed the data, interpreted them, and wrote the manuscript. All authors contributed to the article and approved the submitted version.

## Conflict of Interest

The authors declare that the research was conducted in the absence of any commercial or financial relationships that could be construed as a potential conflict of interest.

## Publisher’s Note

All claims expressed in this article are solely those of the authors and do not necessarily represent those of their affiliated organizations, or those of the publisher, the editors and the reviewers. Any product that may be evaluated in this article, or claim that may be made by its manufacturer, is not guaranteed or endorsed by the publisher.

## Footnotes

1
https://david.ncifcrf.gov/tools.jsp

2
http://pantherdb.org/

3
https://string-db.org/

4
http://www.heatmapper.ca/expression/
