# Supplementary material for: HigB1 Toxin in Mycobacterium tuberculosis Is Upregulated During Stress and Required to Establish Infection in Guinea Pigs
Source: Front Microbiol. 2021 Nov 30;12:748890. doi: 10.3389/fmicb.2021.748890 (PMC8669151; doi:10.3389/fmicb.2021.748890)
Supplement: Supplementary file 1 [file Table_1.DOCX]

| Drugs | Isoniazid | Rifampicin | Levofloxacin | Ethambutol |
| --- | --- | --- | --- | --- |
| H37Rv | 0.19 µM | 1.9 nM | 0.39 µM | 3.125 µM |
| Δ*higB*1 | 0.19 µM | 1.9nM -3.8 nM | 0.39 µM | 3.125 µM |
| Δ*higB*1-CT | 0.19 µM | 1.9 nM | 0.39 µM | 3.125 µM |

**Table S1**: MIC_99_ value (Minimum Inhibitory Concentration) of the H37Rv wild type, Δ*higB*1 mutant and Δ*higB*1-CT strains were determined upon exposure to various anti-tubercular drugs.
