# Supplementary material for: HigB1 Toxin in Mycobacterium tuberculosis Is Upregulated During Stress and Required to Establish Infection in Guinea Pigs
Source: Front Microbiol. 2021 Nov 30;12:748890. doi: 10.3389/fmicb.2021.748890 (PMC8669151; doi:10.3389/fmicb.2021.748890)
Supplement: Supplementary file 2 [file Table_2.DOCX]

| **Locus Tag** | **Gene Name** | **Microarray data for complemented strain compared to wild type of *M. tuberculosis* H37Rv** |
| --- | --- | --- |
|  |  |  |
|  |  | **Fold regulated** |
| Rv0001 | dnaA | 2.12 ↑ |
| Rv0007 | Rv0007 | 2.13 ↑ |
| Rv0013 | trpG | 2.04 ↑ |
| Rv0023 | Rv0023 | 2.62 ↓ |
| Rv0027 | Rv0027 | 2.00 ↓ |
| Rv0039c | Rv0039c | 2.01 ↑ |
| Rv0040c | mtc28 | 3.25 ↑ |
| Rv0042c | Rv0042c | 2.02 ↓ |
| Rv0045c | Rv0045c | 2.12 ↑ |
| Rv0046c | ino1 | 4.20 ↑ |
| Rv0047c | Rv0047c | 5.69 ↑ |
| Rv0053 | rpsF | 2.86 ↑ |
| Rv0063 | Rv0063 | 2.20 ↑ |
| Rv0066c | icd2 | 2.22 ↓ |
| Rv0067c | Rv0067c | 2.85 ↓ |
| Rv0078A | Rv0078A | 2.10 ↓ |
| Rv0079 | Rv0079 | 13.69 ↓ |
| Rv0080 | Rv0080 | 16.74 ↓ |
| Rv0081 | Rv0081 | 3.71 ↓ |
| Rv0101 | nrp | 2.03 ↓ |
| Rv0104 | Rv0104 | 2.61 ↑ |
| Rv0118c | oxcA | 3.90 ↓ |
| Rv0119 | fadD7 | 2.27 ↓ |
| Rv0124 | PE_PGRS2 | 2.37 ↑ |
| Rv0129c | fbpC | 2.04 ↑ |
| Rv0145 | Rv0145 | 2.31 ↑ |
| Rv0166 | fadD5 | 2.09 ↑ |
| Rv0167 | yrbE1A | 2.03 ↑ |
| Rv0168 | yrbE1B | 2.47 ↑ |
| Rv0169 | mce1A | 2.09 ↑ |
| Rv0177 | Rv0177 | 2.34 ↑ |
| Rv0190 | Rv0190 | 2.69 ↑ |
| Rv0196 | Rv0196 | 11.25 ↓ |
| Rv0197 | Rv0197 | 5.57 ↓ |

| **Locus Tag** | **Gene Name** | **Microarray data for complemented strain compared to wild type of *M.tuberculosis* H37Rv** |
| --- | --- | --- |
|  |  | **Fold regulated** |
| Rv0207c | Rv0207c | 2.01 ↑ |
| Rv0211 | pckA | 2.95 ↓ |
| Rv0244c | fadE5 | 5.54 ↓ |
| Rv0278c | PE_PGRS3 | 3.23 ↑ |
| Rv0279c | PE_PGRS4 | 5.14 ↑ |
| Rv0280 | PPE3 | 8.68 ↑ |
| Rv0281 | Rv0281 | 4.67 ↑ |
| Rv0282 | Rv0282 | 2.79 ↑ |
| Rv0286 | PPE4 | 3.20 ↑ |
| Rv0288 | esxH | 2.83 ↑ |
| Rv0289 | Rv0289 | 2.60 ↑ |
| Rv0290 | Rv0290 | 2.13 ↑ |
| Rv0291 | mycP3 | 2.70 ↑ |
| Rv0292 | Rv0292 | 2.52 ↑ |
| Rv0297 | PE_PGRS5 | 2.07 ↑ |
| Rv0307c | Rv0307c | 3.01 ↓ |
| Rv0308 | Rv0308 | 2.09 ↓ |
| Rv0311 | Rv0311 | 4.17 ↓ |
| Rv0312 | Rv0312 | 2.22 ↑ |
| Rv0315 | Rv0315 | 2.37 ↑ |
| Rv0346c | ansP2 | 2.31 ↑ |
| Rv0348 | Rv0348 | 2.23 ↓ |
| Rv0385 | Rv0385 | 2.26 ↑ |
| Rv0395 | Rv0395 | 2.11 ↓ |
| Rv0421c | Rv0421c | 2.14 ↑ |
| Rv0467 | icl | 2.77 ↓ |
| Rv0483 | lprQ | 3.00 ↓ |
| Rv0485 | Rv0485 | 2.11 ↓ |
| Rv0503c | cmaA2 | 2.21 ↑ |
| Rv0524 | hemL | 2.03 ↓ |
| Rv0525 | Rv0525 | 2.39 ↓ |
| Rv0532 | PE_PGRS6 | 2.44 ↑ |
| Rv0535 | pnp | 2.00 ↑ |
| Rv0547c | Rv0547c | 2.31 ↓ |
| Rv0549c | Rv0549c | 2.06 ↓ |
| Rv0558 | ubiE | 2.18 ↓ |
| Rv0569 | Rv0569 | 5.11 ↓ |
| Rv0570 | nrdZ | 3.00 ↓ |
| Rv0571c | Rv0571c | 3.05 ↓ |
| Rv0572c | Rv0572c | 3.59 ↓ |

| **Locus Tag** | **Gene Name** | **Microarray data for complemented strain compared to wild type of *M.tuberculosis* H37Rv** |
| --- | --- | --- |
|  |  | **Fold regulated** |
| Rv0573c | Rv0573c | 2.90 ↓ |
| Rv0574c | Rv0574c | 2.97 ↓ |
| Rv0578c | PE_PGRS7 | 2.25 ↑ |
| Rv0603 | Rv0603 | 2.65 ↑ |
| Rv0609 | Rv0609 | 2.54 ↓ |
| Rv0620 | galK | 2.90 ↑ |
| Rv0625c | Rv0625c | 2.02 ↑ |
| Rv0634c | Rv0634c | 2.31 ↓ |
| Rv0643c | mmaA3 | 2.26 ↑ |
| Rv0648 | Rv0648 | 4.78 ↑ |
| Rv0649 | fabD2 | 3.79 ↑ |
| Rv0650 | Rv0650 | 2.86 ↑ |
| Rv0651 | rplJ | 2.57 ↑ |
| Rv0652 | rplL | 2.67 ↑ |
| Rv0678 | Rv0678 | 3.07 ↓ |
| Rv0691c | Rv0691c | 2.36 ↓ |
| Rv0692 | Rv0692 | 3.66 ↓ |
| Rv0693 | pqqE | 3.09 ↓ |
| Rv0694 | lldD1 | 3.11 ↓ |
| Rv0700 | rpsJ | 2.21 ↑ |
| Rv0746 | PE_PGRS9 | 4.70 ↑ |
| Rv0747 | PE_PGRS10 | 3.36 ↑ |
| Rv0759c | Rv0759c | 2.02 ↑ |
| Rv0787 | Rv0787 | 2.45 ↓ |
| Rv0816c | thiX | 2.82 ↑ |
| Rv0823c | Rv0823c | 2.00 ↑ |
| Rv0825c | Rv0825c | 2.26 ↑ |
| Rv0833 | PE_PGRS13 | 4.31 ↑ |
| Rv0847 | lpqS | 13.18 ↑ |
| Rv0848 | cysK2 | 7.95 ↑ |
| Rv0849 | Rv0849 | 2.39 ↑ |
| Rv0850 | Rv0850 | 2.85 ↑ |
| Rv0867c | rpfA | 2.24 ↓ |
| Rv0886 | fprB | 2.18 ↑ |
| Rv0888 | Rv0888 | 3.25 ↑ |
| Rv0896 | gltA | 2.17 ↓ |
| Rv0914c | Rv0914c | 2.77 ↓ |
| Rv0951 | sucC | 2.35 ↓ |

| **Locus Tag** | **Gene Name** | **Microarray data for complemented strain compared to wild type of *M.tuberculosis* H37Rv** |
| --- | --- | --- |
|  |  | **Fold regulated** |
| Rv0952 | sucD | 2.29 ↓ |
| Rv0967 | Rv0967 | 3.35 ↑ |
| Rv0968 | Rv0968 | 4.57 ↑ |
| Rv0969 | ctpV | 3.00 ↑ |
| Rv0980c | PE_PGRS18 | 2.39 ↑ |
| Rv0993 | galU | 2.12 ↑ |
| Rv1014c | pth | 2.45 ↑ |
| Rv1018c | glmU | 2.22 ↑ |
| Rv1057 | Rv1057 | 4.57 ↓ |
| Rv1063c | Rv1063c | 2.36 ↓ |
| Rv1067c | PE_PGRS19 | 4.60 ↑ |
| Rv1091 | PE_PGRS22 | 2.37 ↑ |
| Rv1092c | coaA | 2.09 ↓ |
| Rv1095 | phoH2 | 2.48 ↑ |
| Rv1129c | Rv1129c | 3.03 ↓ |
| Rv1130 | Rv1130 | 11.13 ↓ |
| Rv1131 | gltA1 | 8.49 ↓ |
| Rv1133c | metE | 2.07 ↑ |
| Rv1148c | Rv1148c | 3.99 ↓ |
| Rv1157c | Rv1157c | 4.18 ↑ |
| Rv1158c | Rv1158c | 3.16 ↑ |
| Rv1159A | phhB | 2.63 ↓ |
| Rv1171 | Rv1171 | 2.01 ↑ |
| Rv1172c | PE12 | 2.61 ↑ |
| Rv1179c | Rv1179c | 2.23 ↑ |
| Rv1184c | Rv1184c | 2.29 ↑ |
| Rv1213 | glgC | 2.15 ↓ |
| Rv1216c | Rv1216c | 3.45 ↓ |
| Rv1217c | Rv1217c | 2.37 ↓ |
| Rv1218c | Rv1218c | 4.17 ↓ |
| Rv1219c | Rv1219c | 2.88 ↓ |
| Rv1230c | Rv1230c | 2.02 ↑ |
| Rv1243c | PE_PGRS23 | 2.95 ↑ |
| Rv1286 | cysN | 2.01 ↓ |
| Rv1289 | Rv1289 | 2.23 ↓ |
| Rv1297 | rho | 2.07 ↑ |
| **Locus Tag** | **Gene Name** | **Microarray data for complemented strain compared to wild type of *M. tuberculosis* H37Rv** |
|  |  | **Fold regulated** |
| Rv1303 | Rv1303 | 2.33 ↓ |
| Rv1304 | atpB | 2.18 ↓ |
| Rv1305 | atpE | 3.51 ↓ |
| Rv1306 | atpF | 3.60 ↓ |
| Rv1307 | atpH | 2.87 ↓ |
| Rv1308 | atpA | 2.80 ↓ |
| Rv1309 | atpG | 2.90 ↓ |
| Rv1310 | atpD | 2.26 ↓ |
| Rv1325c | PE_PGRS24 | 3.28 ↑ |
| Rv1361c | PPE19 | 54.86 ↑ |
| Rv1396c | PE_PGRS25 | 3.61 ↑ |
| Rv1435c | Rv1435c | 2.12 ↑ |
| Rv1441c | PE_PGRS26 | 2.99 ↑ |
| Rv1450c | PE_PGRS27 | 2.65 ↑ |
| Rv1451 | ctaB | 2.03 ↓ |
| Rv1452c | PE_PGRS28 | 3.18 ↑ |
| Rv1456c | Rv1456c | 2.21 ↓ |
| Rv1474c | Rv1474c | 2.04 ↓ |
| Rv1477 | Rv1477 | 3.06 ↑ |
| Rv1478 | Rv1478 | 3.20 ↑ |
| Rv1502 | Rv1502 | 2.20 ↓ |
| Rv1535 | Rv1535 | 4.17 ↑ |
| Rv1540 | Rv1540 | 2.01 ↓ |
| Rv1553 | frdB | 2.48 ↑ |
| Rv1600 | hisC1 | 2.06 ↓ |
| Rv1607 | chaA | 2.02 ↓ |
| Rv1620c | cydC | 3.66 ↓ |
| Rv1621c | cydD | 3.13 ↓ |
| Rv1622c | cydB | 8.29 ↓ |
| Rv1623c | cydA | 6.83 ↓ |
| Rv1632c | Rv1632c | 3.05 ↑ |
| Rv1651c | PE_PGRS30 | 2.12 ↑ |
| Rv1652 | argC | 2.81 ↑ |
| Rv1679 | fadE16 | 2.10 ↑ |
| Rv1733c | Rv1733c | 2.38 ↓ |
| Rv1734c | Rv1734c | 2.69 ↓ |
| Rv1737c | narK2 | 5.40 ↓ |
| Rv1738 | Rv1738 | 7.49 ↓ |
| Rv1739c | Rv1739c | 3.96 ↓ |
| Rv1754c | Rv1754c | 2.44 ↑ |

| **Locus Tag** | **Gene Name** | **Microarray data for complemented strain compared to wild type of *M. tuberculosis* H37Rv** |
| --- | --- | --- |
|  |  | **Fold regulated** |
| Rv1755c | plcD | 11.53 ↓ |
| Rv1759c | wag22 | 3.75 ↑ |
| Rv1768 | PE_PGRS31 | 2.69 ↑ |
| Rv1778c | Rv1778c | 4.79 ↓ |
| Rv1779c | Rv1779c | 9.01 ↓ |
| Rv1815 | Rv1815 | 4.46 ↑ |
| Rv1816 | Rv1816 | 5.60 ↑ |
| Rv1818c | PE_PGRS33 | 2.24 ↑ |
| Rv1831 | Rv1831 | 2.28 ↑ |
| Rv1840c | PE_PGRS34 | 3.32 ↑ |
| Rv1846c | Rv1846c | 2.57 ↓ |
| Rv1854c | ndh | 4.17 ↓ |
| Rv1860 | apa | 2.52 ↑ |
| Rv1878 | glnA3 | 2.00 ↓ |
| Rv1882c | Rv1882c | 2.48 ↑ |
| Rv1883c | Rv1883c | 2.31 ↑ |
| Rv1884c | rpfC | 2.88 ↑ |
| Rv1904 | Rv1904 | 2.34 ↑ |
| Rv1922 | Rv1922 | 2.86 ↓ |
| Rv1952 | Rv1952 | 2.12 ↑ |
| Rv1954c | Rv1954c | 2.38 ↑ |
| Rv1955 | Rv1955 | 5.32 ↑ |
| Rv1982c | Rv1982c | 2.26 ↓ |
| Rv1996 | Rv1996 | 5.15 ↓ |
| Rv2005c | Rv2005c | 6.05 ↓ |
| Rv2032 | acg | 5.16 ↓ |
| Rv2052c | Rv2052c | 2.69 ↑ |
| Rv2053c | fxsA | 2.22 ↑ |
| Rv2056c | rpsN | 3.44 ↑ |
| Rv2057c | rpmG | 3.32 ↑ |
| Rv2058c | rpmB | 5.76 ↑ |
| Rv2059 | Rv2059 | 3.03 ↑ |
| Rv2069 | sigC | 2.28 ↓ |
| Rv2075c | Rv2075c | 3.05 ↑ |
| Rv2122c | hisE | 2.52 ↓ |
| Rv2153c | murG | 2.25 ↑ |
| Rv2157c | murF | 2.67 ↑ |

| **Locus Tag** | **Gene Name** | **Microarray data for complemented strain compared to wild type of *M.tuberculosis* H37Rv** |
| --- | --- | --- |
|  |  | **Fold regulated** |
| Rv2158c | murE | 3.05 ↑ |
| Rv2160A | Rv2160A | 2.26 ↑ |
| Rv2161c | Rv2161c | 2.19 ↑ |
| Rv2164c | Rv2164c | 2.11 ↑ |
| Rv2165c | mraW | 2.02 ↑ |
| Rv2166c | Rv2166c | 2.15 ↑ |
| Rv2180c | Rv2180c | 2.37 ↓ |
| Rv2193 | ctaE | 2.14 ↓ |
| Rv2195 | qcrA | 2.61 ↓ |
| Rv2196 | qcrB | 2.10 ↓ |
| Rv2217 | lipB | 2.16 ↓ |
| Rv2234 | ptpA | 2.06 ↓ |
| Rv2246 | kasB | 2.46 ↑ |
| Rv2248 | Rv2248 | 2.42 ↑ |
| Rv2251 | Rv2251 | 2.36 ↑ |
| Rv2254c | Rv2254c | 2.32 ↑ |
| Rv2255c | Rv2255c | 2.23 ↑ |
| Rv2271 | Rv2271 | 2.37 ↓ |
| Rv2280 | Rv2280 | 2.46 ↓ |
| Rv2309c | Rv2309c | 3.41 ↓ |
| Rv2327 | Rv2327 | 2.88 ↓ |
| Rv2358 | Rv2358 | 2.00 ↓ |
| Rv2391 | nirA | 2.60 ↑ |
| Rv2392 | cysH | 2.28 ↑ |
| Rv2396 | PE_PGRS41 | 3.39 ↑ |
| Rv2401A | Rv2401A | 2.06 ↑ |
| Rv2406c | Rv2406c | 2.62 ↑ |
| Rv2407 | Rv2407 | 2.01 ↓ |
| Rv2427A | Rv2427A | 2.11 ↓ |
| Rv2428 | ahpC | 4.77 ↓ |
| Rv2429 | ahpD | 3.06 ↓ |
| Rv2440c | obgE | 2.01 ↑ |
| Rv2450c | rpfE | 2.06 ↓ |
| Rv2452c | Rv2452c | 2.28 ↓ |
| Rv2454c | Rv2454c | 2.20 ↓ |
| Rv2455c | Rv2455c | 2.24 ↓ |
| Rv2456c | Rv2456c | 2.32 ↑ |
| Rv2461c | clpP | 2.27 ↓ |
| Rv2462c | tig | 3.12 ↓ |
| Rv2463 | lipP | 16.15↓ |

| **Locus Tag** | **Gene Name** | **Microarray data for complemented strain compared to wild type of *M. tuberculosis* H37Rv** |
| --- | --- | --- |
|  |  | **Fold regulated** |
| Rv2480c | Rv2480c | 2.06 ↓ |
| Rv2485c | lipQ | 2.42 ↑ |
| Rv2487c | PE_PGRS42 | 2.50 ↑ |
| Rv2489c | Rv2489c | 3.14 ↑ |
| Rv2524c | fas | 2.46 ↑ |
| Rv2541 | Rv2541 | 4.36 ↑ |
| Rv2591 | PE_PGRS44 | 2.84 ↑ |
| Rv2592c | ruvB | 2.19 ↑ |
| Rv2627c | Rv2627c | 9.55 ↓ |
| Rv2628 | Rv2628 | 10.84 ↓ |
| Rv2629 | Rv2629 | 5.00 ↓ |
| Rv2633c | Rv2633c | 2.08 ↑ |
| Rv2638 | Rv2638 | 2.06 ↑ |
| Rv2705c | Rv2705c | 2.32 ↓ |
| Rv2706c | Rv2706c | 3.27 ↓ |
| Rv2721c | Rv2721c | 2.17 ↑ |
| Rv2729c | Rv2729c | 2.03 ↓ |
| Rv2799 | Rv2799 | 2.34 ↑ |
| Rv2944 | Rv2944 | 2.42 ↑ |
| Rv2950c | fadD29 | 2.11 ↑ |
| Rv2954c | Rv2954c | 2.17 ↑ |
| Rv2980 | Rv2980 | 2.06 ↑ |
| Rv2985 | mutT1 | 2.06 ↑ |
| Rv2986c | hupB | 2.10 ↑ |
| Rv2987c | leuD | 2.92 ↑ |
| Rv2988c | leuC | 2.87 ↑ |
| Rv2990c | Rv2990c | 7.49 ↑ |
| Rv3016 | lpqA | 2.72 ↓ |
| Rv3023c | Rv3023c | 2.01 ↑ |
| Rv3027c | Rv3027c | 2.09 ↑ |
| Rv3048c | nrdF2 | 2.55 ↑ |
| Rv3054c | Rv3054c | 2.19 ↓ |
| Rv3083 | Rv3083 | 3.02 ↓ |
| Rv3084 | lipR | 2.23 ↓ |
| Rv3085 | Rv3085 | 2.89 ↓ |
| Rv3086 | adhD | 2.55 ↓ |
| Rv3087 | Rv3087 | 2.70 ↓ |
| Rv3088 | Rv3088 | 2.54 ↓ |

| **Locus Tag** | **Gene Name** | **Microarray data for complemented strain compared to wild type of *M.tuberculosis* H37Rv** |
| --- | --- | --- |
|  |  | **Fold regulated** |
| Rv3089 | fadD13 | 2.67 ↓ |
| Rv3127 | Rv3127 | 9.85 ↓ |
| Rv3129 | Rv3129 | 5.57 ↓ |
| Rv3130c | tgs1 | 11.40 ↓ |
| Rv3131 | Rv3131 | 3.71 ↓ |
| Rv3134c | Rv3134c | 4.83 ↓ |
| Rv3135 | PPE50 | 3.13 ↑ |
| Rv3136 | PPE51 | 2.50 ↑ |
| Rv3139 | fadE24 | 3.49 ↓ |
| Rv3140 | fadE23 | 2.62 ↓ |
| Rv3161c | Rv3161c | 2.39 ↓ |
| Rv3171c | hpx | 2.00 ↓ |
| Rv3228 | Rv3228 | 2.41 ↑ |
| Rv3229c | Rv3229c | 2.61 ↑ |
| Rv3260c | whiB2 | 2.39 ↑ |
| Rv3269 | Rv3269 | 2.58 ↓ |
| Rv3295 | Rv3295 | 2.01 ↑ |
| Rv3312A | Rv3312A | 3.33 ↑ |
| Rv3330 | dacB1 | 2.90 ↑ |
| Rv3331 | sugI | 2.77 ↑ |
| Rv3332 | nagA | 2.59 ↑ |
| Rv3339c | icd1 | 2.15 ↓ |
| Rv3344c | PE_PGRS49 | 3.07 ↑ |
| Rv3345c | PE_PGRS50 | 5.62 ↑ |
| Rv3367 | PE_PGRS51 | 2.73 ↑ |
| Rv3388 | PE_PGRS52 | 2.68 ↑ |
| Rv3390 | lpqD | 3.07 ↑ |
| Rv3391 | acrA1 | 2.45 ↑ |
| Rv3408 | Rv3408 | 2.13 ↓ |
| Rv3413c | Rv3413c | 2.01 ↑ |
| Rv3428c | Rv3428c | 3.82 ↓ |
| Rv3469c | mhpE | 3.59 ↓ |
| Rv3478 | PPE60 | 302.53 ↓ |
| Rv3486 | Rv3486 | 2.00 ↑ |
| Rv3487c | lipF | 2.35 ↑ |
| Rv3500c | yrbE4B | 2.01 ↑ |
| Rv3507 | PE_PGRS53 | 4.84 ↑ |
| Rv3508 | PE_PGRS54 | 4.73 ↑ |

| **Locus Tag** | **Gene Name** | **Microarray data for complemented strain compared to wild type of *M.tuberculosis* H37Rv** |
| --- | --- | --- |
|  |  | **Fold regulated** |
| Rv3511 | PE_PGRS55 | 9.35 ↑ |
| Rv3513c | fadD18 | 4.00 ↑ |
| Rv3514 | PE_PGRS57 | 3.03 ↑ |
| Rv3572 | Rv3572 | 2.14 ↑ |
| Rv3587c | Rv3587c | 2.12 ↑ |
| Rv3590c | PE_PGRS58 | 2.48 ↑ |
| Rv3595c | PE_PGRS59 | 2.05 ↑ |
| Rv3611 | Rv3611 | 2.34 ↑ |
| Rv3612c | Rv3612c | 2.38 ↑ |
| Rv3613c | Rv3613c | 3.60 ↑ |
| Rv3614c | Rv3614c | 3.13 ↑ |
| Rv3615c | Rv3615c | 3.73 ↑ |
| Rv3616c | Rv3616c | 2.50 ↑ |
| Rv3623 | lpqG | 2.05 ↑ |
| Rv3645 | Rv3645 | 2.20 ↓ |
| Rv3651 | Rv3651 | 2.25 ↓ |
| Rv3660c | Rv3660c | 2.27 ↑ |
| Rv3662c | Rv3662c | 3.52 ↑ |
| Rv3663c | dppD | 2.53 ↑ |
| Rv3686c | Rv3686c | 2.96 ↑ |
| Rv3699 | Rv3699 | 2.47 ↓ |
| Rv3705c | Rv3705c | 2.25 ↑ |
| Rv3712 | Rv3712 | 2.14 ↑ |
| Rv3717 | Rv3717 | 2.26 ↑ |
| Rv3739c | PPE67 | 5.85 ↑ |
| Rv3743c | ctpJ | 8.95 ↑ |
| Rv3750c | Rv3750c | 2.49 ↑ |
| Rv3811 | Rv3811 | 2.40 ↑ |
| Rv3822 | Rv3822 | 2.10 ↑ |
| Rv3825c | pks2 | 2.88 ↑ |
| Rv3848 | Rv3848 | 2.44 ↓ |
| Rv3918c | parA | 2.14 ↑ |

**Table S2: Genes showing differential expression of > 2.0-fold in Rv1955 complemented strain vs Wild type strain**
